# Penehyclidine hydrochloride alleviates lung ischemia-reperfusion injury by inhibiting pyroptosis

**DOI:** 10.1186/s12890-024-03018-5

**Published:** 2024-04-26

**Authors:** Rongfang Liu, Xuguang Zhang, Jing Yan, Shan Liu, Yongle Li, Guangyi Wu, Jingui Gao

**Affiliations:** 1 Department of Anesthesiology, the Second Hospital of Hebei Medical University, NO. 215 of HePing West Road, Xinhua District Shijiazhuang, 050000 Shijiazhuang, China; 2 Department of Anesthesiology, Affiliated Hospital of Hebei University, 071000 Baoding, China; 3 Department of Thoracic surgery, Affiliated Hospital of Hebei University, 071000 Baoding, China; 4 Electron microscope room, Hebei Medical University, 050000 Shijiazhuang, China; 5 Department of Pathology, Affiliated Hospital of Hebei University, 071000 Baoding, China

**Keywords:** Inflammatory response, Left hilum clamp, Lung ischemia-reperfusion injury, Oxidative stress, Penehyclidine hydrochloride, Pyroptosis

## Abstract

**Objective:**

The aim of this research was to examine how penehyclidine hydrochloride (PHC) impacts the occurrence of pyroptosis in lung tissue cells within a rat model of lung ischemia-reperfusion injury.

**Methods:**

Twenty-four Sprague Dawley (SD) rats, weighing 250 g to 270 g, were randomly distributed into three distinct groups as outlined below: a sham operation group (S group), a control group (C group), and a test group (PHC group). Rats in the PHC group received a preliminary intravenous injection of PHC at a dose of 3 mg/kg. At the conclusion of the experiment, lung tissue and blood samples were collected and properly stored for subsequent analysis. The levels of malondialdehyde, superoxide dismutase, and myeloperoxidase in the lung tissue, as well as IL-18 and IL-1β in the blood serum, were assessed using an Elisa kit. Pyroptosis-related proteins, including Caspase1 p20, GSDMD-N, and NLRP3, were detected through the western blot method. Additionally, the dry-to-wet ratio (D/W) of the lung tissue and the findings from the blood gas analysis were also documented.

**Results:**

In contrast to the control group, the PHC group showed enhancements in oxygenation metrics, reductions in oxidative stress and inflammatory reactions, and a decrease in lung injury. Additionally, the PHC group exhibited lowered levels of pyroptosis-associated proteins, including the N-terminal segment of gasdermin D (GSDMD-N), caspase-1p20, and nucleotide-binding oligomerization domain-like receptor protein 3 (NLRP3).

**Conclusion:**

Pre-administration of PHC has the potential to mitigate lung ischemia-reperfusion injuries by suppressing the pyroptosis of lung tissue cells, diminishing inflammatory reactions, and enhancing lung function. The primary mechanism behind anti-pyroptotic effect of PHC appears to involve the inhibition of oxidative stress.

**Supplementary Information:**

The online version contains supplementary material available at 10.1186/s12890-024-03018-5.

## Introduction

Lung ischemia-reperfusion injury (IRI), a type of aseptic acute lung injury, is a common surgical complication following single-lung ventilation [1, 2], open-heart surgery [3], trauma [4], and lung transplantation [5]. The endothelium layer of the pulmonary vasculature produces reactive oxygen species during the process, which triggers a cascade reaction that ultimately results in pulmonary dysfunction, even respiratory failure, and increases patient mortality. The complex process of lung ischemia-reperfusion involves a number of pathogenic processes and possible mechanisms, including autophagy [6], apoptosis [7], pyroptosis [8], and oxidative stress injury [9]. As a result, many medications or other substances connected to the aforementioned pathological processes are used to treat lung ischemia-reperfusion injury, such as dexmedetomidine [10], hydrogen [5], ulinastatin [11], pirfenidone [12], and penehyclidine hydrochloride (PHC) [13]. PHC, a brand-new anticholinergic medication derived from scopolamine that selectively blocks M1 and M3 muscarinic receptors, has strong peripheral and cerebral anticholinergic actions [14]. PHC can rapidly cross the blood-brain barrier and has little cardiovascular adverse effects related to the M2 receptor. PHC may have protective benefits on a number of organs, including the kidney [15], brain [16], lung [17], and heart [18]. PHC also has a significant impact on the management of chronic obstructive pulmonary disease (COPD) by reducing the activity of toll-like receptors and by reducing the pulmonary inflammatory response brought on by mechanical breathing in COPD rats [19]. Additionally, due to its capacity to raise beta-arrestin-120, PHC provides a protective effect against pulmonary injury brought on by lipopolysaccharides [20] Additionally, PHC pretreatment prior to ischemia-reperfusion may decrease lung injury in rats [13]. Further research is still needed to determine the precise processes underpinning the ability of PHC to reduce lung IRI.

## Materials and methods

### Animals

Adult male Sprague-Dawley rats, weighing between 250 and 270 g, were procured from Beijing Vital River Laboratory Animal Technology. They were accommodated in specialized cages equipped with ample food and water supply and maintained in a controlled environment with a 12-hour light-dark cycle. Ethical approval for this experiment was granted by the Animal Care and Use Committee of Hebei University.

A total of 24 rats were randomly assigned to one of three groups: the sham operation group (referred to as the S group), the control group (referred to as the C group), and the PHC pretreatment group (referred to as the PHC group). Rats in the S group underwent thoracotomy, where the hilum structure was carefully separated and fully exposed without any clamping. In contrast, rats in the other two groups had their left hilum clamped using a non-invasive vascular clamp to induce an ischemic model.

### Rat lung ischemia-reperfusion model

All the rats were rendered unconscious through an intraperitoneal injection of 5 mg/100 g of pentobarbital sodium. Once they were adequately anesthetized, they were placed on an insulating blanket and had a tracheotomy performed for intubation. A tracheal tube was then connected to a small animal ventilator (Rayward, R415, Shenzhen, China) and ventilated with 100% oxygen. The tidal volume used was 6–8 ml per kilogram of body weight, following a volume-control mode, at a rate of 60–80 breaths per minute, with an inhalation-to-exhalation ratio of 1:2.

After 30 min of intravenous administration of PHC (3 mg/kg) [13] through the femoral vein, the rats underwent a left thoracotomy, specifically during the expiratory phase, through the fourth intercostal space. Ten minutes later, following intravenous injection of sodium heparin (200 U/kg), the left hilum structure was carefully clamped using a non-invasive vascular clamp. After a duration of 45 min, the vascular clamp was removed, and pulmonary blood perfusion was maintained for a period of 2 h.

Throughout the entire experiment, normal saline was continuously administered through the femoral vein at a rate of 10 ml per kilogram per hour. The blood pressure and temperature were consistently monitored using the Mindray iPM10 equipment from Shenzhen, China. The model rats were euthanized by exsanguination 2 h after reperfusion. Lung tissues and serum samples were collected for subsequent testing.

### Blood gas analysis

A total volume of 1 ml of arterial blood was collected from the femoral artery. Blood gas analysis was conducted at two time points: before the thoracotomy (T0) and either at the conclusion of the experiment or after a 2-hour reperfusion period (T2). This analysis was carried out using the OPTI CCA-TS Blood Gas and Electrolyte Analyzer from the United States. Subsequently, the oxygenation index (PaO2/FiO2) was calculated to assess pulmonary function.

### Sample processing and collection

After concluding the experiment, approximately 2–4 ml of blood were obtained from the femoral artery and subsequently spun at 2000 rpm at 4 °C for 15 min. The resulting supernatant was collected and preserved at -80 °C. The lung tissue was extracted, underwent a brief rinse with a cold (4°C) PBS solution, and was then subdivided into multiple smaller segments using a sharp blade. Some of these segments were promptly placed in a -80 °C freezer after pre-cooling with liquid nitrogen, while others were designated for pathological analysis and determination of the wet/dry weight ratio (W/D).

### Pulmonary tissue wet/dry weight ratio (W/D)

To assess pulmonary edema, the process involved initially using absorbent paper to remove surface moisture from harvested upper lobe lung tissue. This tissue was then promptly weighed to determine its wet weight. Subsequently, it was placed in a 70 °C oven and subjected to a 72-hour baking period to ascertain the dry weight. Ultimately, the W/D weight ratio was computed as a means of assessing pulmonary edema.

### Histopathologic analysis of lung tissue

In summary, the lung tissue underwent a 72-hour immersion in a 4% paraformaldehyde solution, followed by embedding in paraffin and slicing into 4 μm-thick sections. These sections were subsequently stained with hematoxylin and eosin. A pathologist, unaware of the experimental design, examined the histological changes in the pulmonary tissue under a microscope and recorded the lung injury score. The parameters assessed included pulmonary interstitial edema, neutrophil infiltration, alveolar edema, and alveolar congestion [21]. The lung injury scores (LIS) were categorized as: 0 = normal or very mild, 1 = mild, 2 = moderate, 3 = severe.

### Levels of inflammatory factors in serum

The concentrations of interleukin (IL)-1β and IL-18 in the serum were measured using enzyme-linked immunosorbent assay (ELISA) kits (Merck Bell Biotechnology, Wuhan, China) following the manufacturer’s provided instructions.

### Detection of oxidative stress in lung tissue

Myeloperoxidase (MPO) function, the malondialdehyde (MDA) concentration, and superoxide dismutase (SOD) performance in lung tissue were assessed using a dedicated kit from Merck Bell Biotechnology (Wuhan, China). All assessments were conducted following the provided instructions from the manufacturer.

### Detection of pyroptosis-related proteins

In accordance with the manufacturer’s guidelines, lung tissue homogenates were processed for total protein extraction using RIPA lysis buffer (Beyotime Biotechnology, Shanghai, China). The resulting mixture was then subjected to centrifugation at 12,000 rpm at 4 °C for 15 min using a Sigma 3–39 K centrifuge (Germany). The concentration of total proteins was determined by employing a BCA kit (Beyotime Biotechnology, Shanghai, China) through the bicinchoninic acid method. Subsequently, the protein samples were obtained, underwent electrophoresis on a 10% sodium dodecyl sulfate-polyacrylamide gel (SDS-PAGE), and were transferred onto polyvinylidene fluoride (PVDF) membranes. These membranes were subsequently blocked with a 5% stacking gel at room temperature for 40 min. Following this, the membranes were subjected to overnight incubation with primary antibodies targeting NLRP3 (1:1,000, Abcam, Cambridge, USA), GSDMD-N (1:1,000, Cell Signaling Technology, USA), cysteinyl aspartate-specific proteinase (caspase-1) p20 (1:1,000, Biorigin, Beijing, China), and anti-β-actin antibody at 4 °C. After overnight incubation, the membranes were treated with a secondary antibody (1:300, Merck, Wuhan, China) following three washes at room temperature. Finally, the immunological complexes were analyzed using ECL analysis kits (Beyotime Biotechnology, Shanghai, China), and the protein expression levels were quantified using image software.

Transmission electron microscopy (TEM).

To observe the morphology of type II alveolar epithelial cells, an expert who was blind to the study design made an electron microscope section of the lung tissue as soon as possible. The lung tissue was washed with 4 °C PBS solution, cut into small pieces (1 mm^3^), and fixed in 2.5% glutaraldehyde for 2 h at room temperature.

### Statistical analysis

Dates are presented as mean ± standard deviation. Statistical analyses were carried out using IBM SPSS Statistics 20.0 software. Group differences were assessed using a combination of one-way analysis of variance (ANOVA) with the Student’s t-test, and within-group comparisons were made using repeated measures ANOVA. A significance level of *P* < 0.05 was employed to determine statistical significance.

## Results

### Blood gas analysis

We employed the ratio of the fraction of inspired oxygen (FiO2) to the partial pressure of arterial oxygen (PaO2) as an indicator to assess the function of the lungs’ oxygenation system. PaO2/FiO2 was comparatively constant in the sham group. The PaO2/FiO2 in the sham group (428 ± 22.41 mmHg) was significantly higher than that in the control group (302 ± 30.49 mmHg) at the conclusion of the experiment, while the PaO2/FiO2 in the PHC group (343 ± 32.83 mmHg) was significantly higher than that in the control group (*P* < 0.05) (Table 1).

**Table 1:** PaO_2_/FiO_2_, the indicators of the inflammatory response and oxidative stress in groups (mean ± SD, *n* = 8)

	T_0_PaO_2_/FiO_2_(mmHg)	T_2_PaO_2_/FiO_2_(mmHg)	W/D	MPO(pg/ml)	IL-1β(ng/l)	IL-18(pg/ml)	MDA(mmol/l)	SOD(pg/ml)
Sham	435 ± 26.9^∇#^	428 ± 22.41^#^	4.1 ± 0.3^#^	27.50 ± 6.10^#^	22.50 ± 5.55^#^	64.22 ± 13.18^#^	3.42 ± 0.46^#^	46.28 ± 4.94^#^
Control	433 ± 32.35^∆*^	302 ± 30.49^*^	6.4 ± 0.5^*^	49.48 ± 4.95^*^	43.79 ± 7.51^*^	137.84 ± 21.78^*^	7.73 ± 1.23^*^	26.90 ± 8.57^*^
PHC	436 ± 31.5^∆∇*^	343 ± 32.83^*#^	5.2 ± 0.4^*#^	38.55 ± 3.95^*#^	28.78 ± 3.80^*#^	85.34 ± 12.03^*#^	5.22 ± 1.09^*#^	36.54 ± 3.30^*#^

Note: T_0_ PaO_2_/FiO_2_:basic value, ^∆^*P* > 0.05 vs. sham group, ^∇^*P* > 0.05 vs. control group; T_2_ PaO_2_/FiO_2_: values of after 2 h reperfusion; W/D, wet weight (W)/dry weight (D); MPO, myeloperoxidase; IL, Interleukin; MDA, malonaldehyde; SOD, superoxide dismutase. ^*^P < 0.05 vs. sham group, ^#^*P* < 0.05 vs. control group

### Inflammatoryresponse

While the W/D ratio of the lung tissue in the PHC group (5.2 ± 0.4) was lower than that in the control group (6.4 ± 0.5), it was higher than that in the sham group (4.1 ± 0.3). The serum concentrations of IL-18 and IL-1 as well as the lung tissue’s MPO activity showed a tendency that was similar to the W/D (*P* < 0.05) (Table 1).

### Oxidative stress response

The MDA levels in lung tissue in the PHC group (5.22 ± 1.09 mmol/l) and sham group (3.42 ± 0.46 mmol/l) were lower than those in the control group (7.73 ± 1.23 mmol/l) (*P* < 0.05) compared to the control group. Additionally, the MPO levels in lung tissue were greater in the PHC group than in the sham group. In contrast to MDA levels, SOD activity in lung tissue displayed the reverse direction (Table 1).

### Lung injury scores

**Fig. 1:**
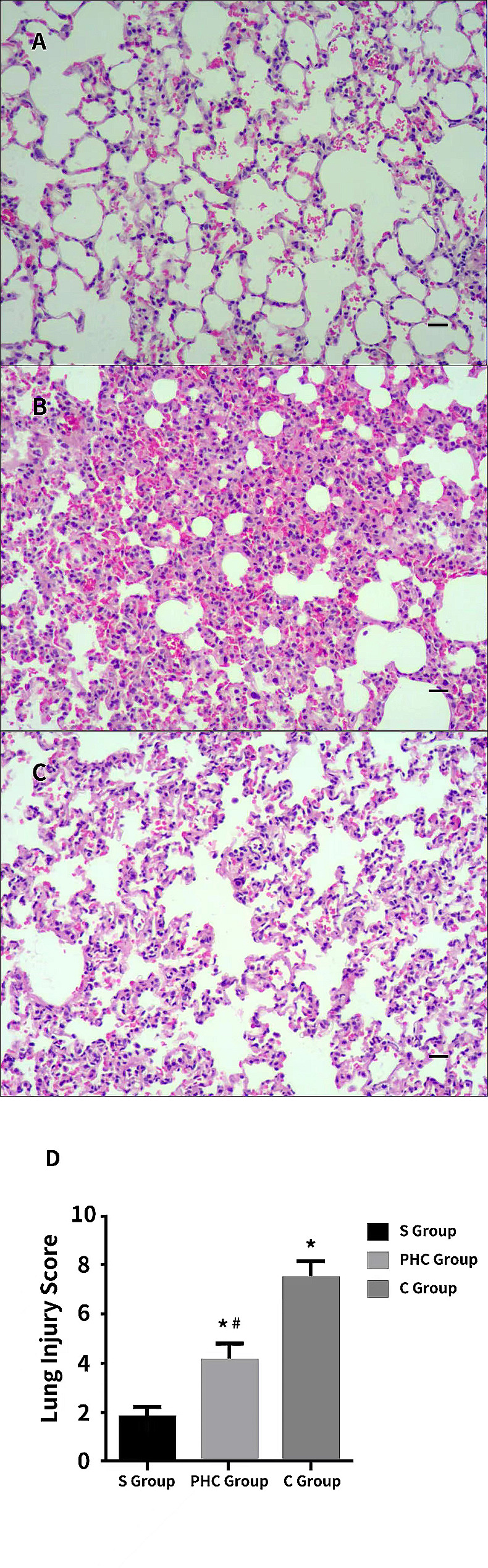
Paraformaldehyde-fixed sections of lung tissues were stained with eosin and hematoxylin, and all pictures represent 40x original magnifications, the scale bar represents 100 μm (black). (**A**) sham group; (**B**) control group; (**C**) PHC group; (**D**) Lung injury score. ^*^P < 0.05 vs. sham group, ^**#**^*P* < 0.05 vs. control group

The sham group exhibited minimal pathological alterations in lung tissue due to the surgical procedure, while the control group displayed a substantial presence of neutrophil infiltration, intra-alveolar hemorrhage, and severe interstitial edema. In comparison to the control group, the PHC group demonstrated fewer pathological changes in lung tissue. The degree of neutrophil infiltration, as indicated by the LIS, was significantly higher in the control group [2.5(2–3)] than in the sham group [0.5(0–1) (*P* < 0.05), and in the PHC group [1.5(1–2)] (*P* < 0.05) (see Fig. 1).

### Pyroptosis-related protein

**Fig. 2:**
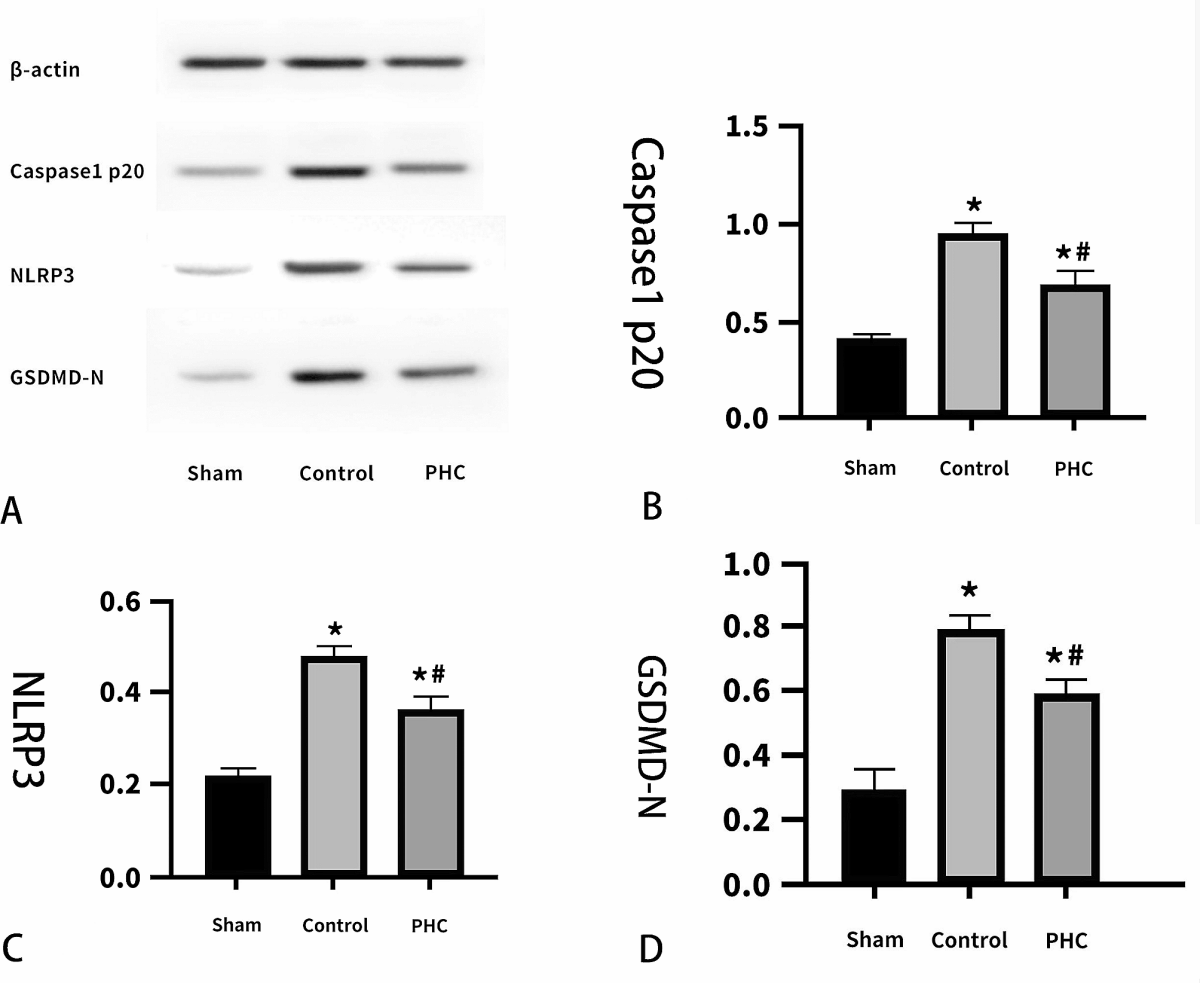
The protein expressions in the lung tissue (*n* = 3/group). (**A**) Western blots, and semi-quantitative analysis of Caspase1-p20 (**B**), NLRP3 (**C**) and GSDMD-N (**D**) ^*^P < 0.05 vs. sham group, ^**#**^*P* < 0.05 vs. control group

Caspase-1 p20 levels in the lung tissues were lower in the sham (0.42 ± 0.2) and PHC groups (0.69 ± 0.75) compared to the control group (0.96 ± 0.51) (*P* < 0.05). Similar trends were seen in the levels of NLRP3 and GSDMD-N as well as Caspase-1 p20 (Fig. 2).

Electron microscopy analysis of morphological changes in type II alveolar epithelial cells.

**Fig. 3:**
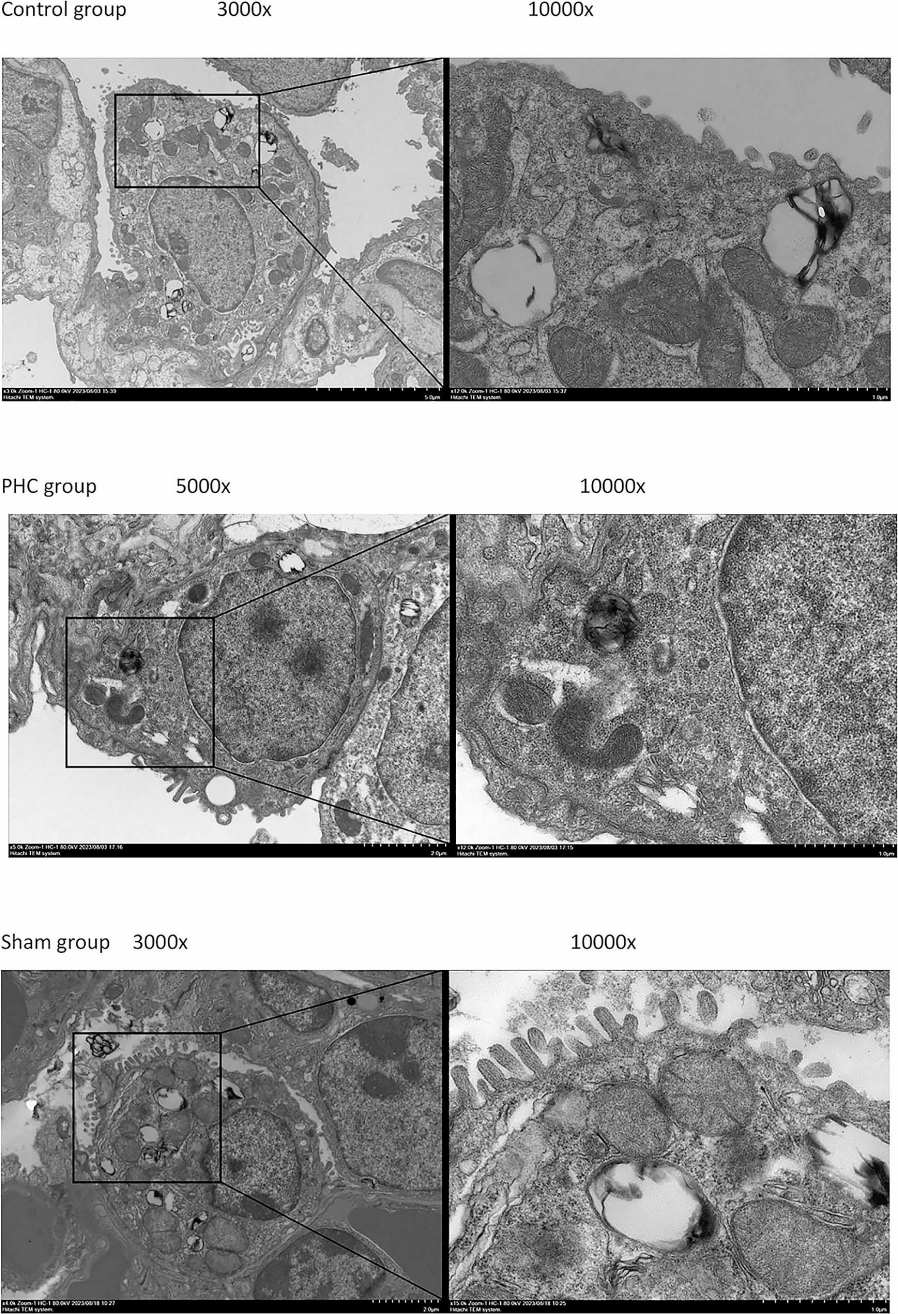
Type II alveolar epithelial cell morphology was imaged with electron microscopy. (Control group), (PHC group), (Sham group), (original magnification, 10,000). As it was shown in the above pictures: in sham group, the type II epithelial alveolar cells were almost normal, villi and lamellar corpuscles were observed easily, with no obvious swelling or destruction of mitochondria. In the control group, the microvilli were slightly shorter, with some parts of the microvilli having reduced or even disappeared. There was a decrease in epithelium villi and lamellar corpuscles, while swollen mitochondria and crista membrane fusion were significant. Additionally, rough endoplasmic reticulum expanded, degranulation occurred, the perinuclear gap was wide and nuclear pyknosis occured, means the lung injury was severe in this group Compared with the control group in the PHC group, there were more epithelium villi and lamellar corpuscles, mitochondria swelling was slighter, crista membrane fusion was decreased and the perinuclear space widen slightly

Ultrastructural alterations in lung tissue were examined using TEM. In the control group, microvilli displayed a slight reduction in length, with certain sections even experiencing a decrease or disappearance. Epithelium villi and lamellar corpuscles exhibited a decline, while swollen mitochondria and the merging of crista membranes were notably prominent. Additionally, the rough endoplasmic reticulum expanded, degranulation was observed, the perinuclear gap widened, and nuclear pyknosis occurred. Comparatively, fewer alterations were observed in the PHC group. However, in the sham group, type II alveolar epithelial cells resembled normal structures with minimal changes. Refer to Fig. 3.

## Discussion

IRI is a pathological condition that arises when endogenous ligands are released [22]. This process occurs in tissues subjected to varying degrees of hypoxia and ischemia caused by various factors. Following the restoration of blood flow, organs and tissues undergo significant oxidative stress, resulting in changes in protein expression, disruptions in cellular metabolism, and structural injury [23]. Consequently, tissue damage is worsened, and in some cases, it becomes irreversible [24]. Lung ischemia-reperfusion injury is a common event during surgical procedures, especially in lung transplantation and cardiothoracic surgery. Nevertheless, the exact underlying mechanism remains unclear. Several theories propose that lung IRI may be linked to cell apoptosis [25], cell autophagy [26], and pyroptosis [8]. In this study, we demonstrated that the administration of PHC prior to ischemia can mitigate the development of lung injury caused by ischemia-reperfusion. This protective effect is associated with the prevention of pyroptosis, which is defined as gasdermin-mediated programmed necrosis and is linked to innate immunity and various diseases [27]. Research has shown a close connection between pyroptosis and conditions such as cardiovascular diseases [28] and tumors [29]. Recent evidence underscores the critical role of pyroptosis in host defense and its primary role in bridging innate and adaptive immunity [30]. However, excessive pyroptosis can lead to prolonged and heightened inflammatory responses, thereby contributing to the progression of inflammatory disorders [31]. 

Consequently, pyroptosis is a two-edged sword and has been observed in various types of cells, including those in the digestive system, reproductive system, cardiovascular system, and central nervous system [32]. Notably, some scholars have also confirmed the involvement of pyroptosis in the mechanism of pulmonary ischemia-reperfusion, aligning with our experimental findings. In the control group, there was an increase in the expression of proteins associated with pyronecrosis, such as NLRP3, caspase-1 p20, and GSDMD-N. Conversely, the expression of pyroptosis-related proteins in the sham group was minimal, and in the PHC group, it fell between the levels observed in the sham and control groups. This suggests that the protective effect of PHC on lung IRI may be linked to the regulation of pyroptosis. Pyroptosis represents an inflammatory type of programmed cell death characterized by cell membrane rupture and a robust inflammatory reaction. In a rat lung ischemia-reperfusion model, levels of pyroptosis-related proteins reached their highest point 2 h after reperfusion [33], which is why a 2-hour reperfusion period was selected for this study. The expression of pyroptosis-related proteins demonstrated that lung IRI is associated with pyroptosis mediated by the NLRP3 inflammasome. There is emerging evidence indicating that PHC offers protection against injuries caused by ischemia-reperfusion in various organs. These include acute lung injury resulting from renal ischemia-reperfusion and acute cerebral ischemia-reperfusion injury. These protective effects are attributed to the activation of the Nrf2 pathway [34] and the suppression of the JNK/p38MAPK signaling pathway [35]. PHC improves conditions related to oxidative stress, apoptosis, and inflammatory responses, effectively preventing renal ischemia-reperfusion [36]. Moreover, PHC elevates PDGF-B levels to activate the PI3K pathway in cells, ameliorating myocardial ischemia-reperfusion injury [37]. 

Furthermore, PHC also exhibits a certain preventive and protective effect against various types of lung injuries. In blunt chest trauma and hemorrhagic shock-induced acute lung injury, PHC plays a crucial role in preventing TLR4 signaling and inflammation [38]. Additionally, PHC has been demonstrated to be beneficial in cases of lung injury resulting from cecal ligation by enhancing microvascular permeability [39]. Research has also shown that PHC can alleviate apoptosis and endoplasmic reticulum stress [40] and trigger the Hes1/Notch1 pathway [41], effectively mitigating the effects of lipopolysaccharide-induced acute lung injury.

Chen et al. speculated that PHC may effectively alleviate lung injury resulting from mechanical ventilation in rats with COPD. The potential mechanism for this process may be related to the JNK/SAPK signaling pathway [19]. In our study, we discovered that pyroptosis is another potential mechanism underlying the protective effect of PHC against lung ischemia-reperfusion injury. Wang et al. [13] determined that the optimal dose of PHC pretreatment in a rat lung ischemia-reperfusion model is 3 mg/kg. Therefore, we used 3 mg/kg of PHC in our experiment and obtained similar results, confirming the significant protective effect of this dosage against lung ischemia-reperfusion injury. Additionally, PHC offers relief from pulmonary injury associated with cardiopulmonary bypass by diminishing the inflammatory response and oxidative stress reaction [42]. In this investigation, prior treatment with PHC demonstrated a significant reduction in inflammation markers, such as IL-18 and IL-1β levels in the bloodstream, MPO activity in lung tissue, and the W/D ratio of the lung.

Moreover, PHC effectively counteracts oxidative stress by reducing MDA activity and enhancing SOD activity, suggesting its potential to exert anti-inflammatory and antioxidant effects by decreasing inflammatory factors and increasing reactive oxygen species activity. These protective properties of PHC imply its capacity to shield against organ ischemia-reperfusion injury.

Nonetheless, our study has several limitations. First, we only examined the injury following a two-hour reperfusion period, necessitating further investigation into the long-term effects of PHC on lung IRI. Second, our observation of pyroptosis in pulmonary ischemia-reperfusion highlights the need for further exploration of the underlying mechanisms, potentially through in vitro cell culture experiments. Third, due to the small sample size and brief reperfusion duration, we were unable to fully record animal mortality rates, leaving uncertainty regarding the potential benefits of PHC in mitigating pulmonary ischemia-reperfusion injury-related mortality.

## Conclusion

Preconditioning with PHC can mitigate lung IRI, as it hinders the pyroptosis of lung tissue cells, diminishes the inflammatory response, and enhances lung function while primarily relying on the inhibition of oxidative stress as its key antipyroptotic mechanism.

### Electronic supplementary material

Below is the link to the electronic supplementary material.

**Supplementary Material 1**: Supplemental figure: The groups of WB gels are: Sham group, Control group, PHC group, Sham group, Control group, PHC group, Sham group, Control group, PHC group

## Data Availability

The datasets used and analysed during the current study are available from the corresponding author on reasonable request.

## Abbreviations

PHC: Penehyclidine hydrochloride
LIS: Lung injury score
LPS: Lipopolysaccharides
COPD: Chronic obstructive pulmonary disease
