# Supplementary figures and images for: Penehyclidine hydrochloride alleviates lung ischemia-reperfusion injury by inhibiting pyroptosis
Source: BMC Pulm Med. 2024 Apr 26;24:207. doi: 10.1186/s12890-024-03018-5 (PMC11046774; doi:10.1186/s12890-024-03018-5)

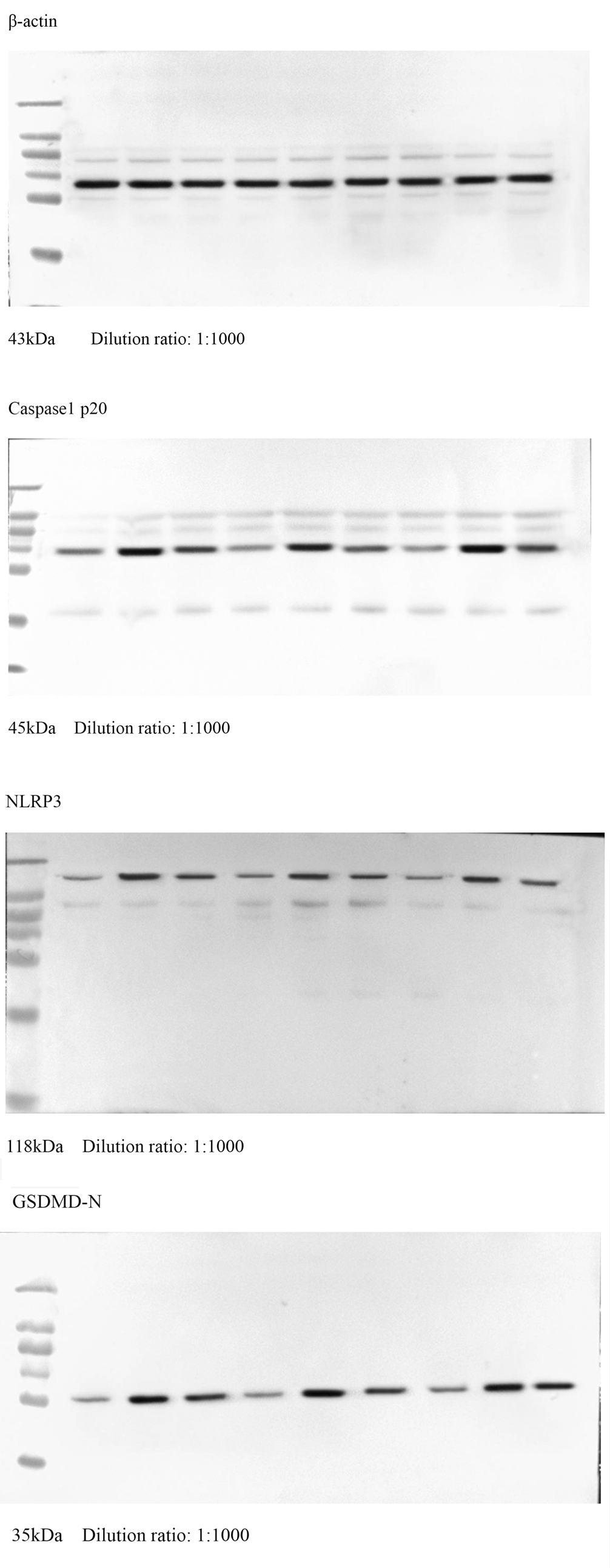

Supplement: Supplementary file 1 — Supplementary Material 1: Supplemental figure: The groups of WB gels are: Sham group, Control group, PHC group, Sham group, Control group, PHC group, Sham group, Control group, PHC group [file 12890_2024_3018_MOESM1_ESM.tif]
